# Picturing of the Lung Tumor Cellular Composition by Multispectral Flow Cytometry

**DOI:** 10.3389/fimmu.2022.827719

**Published:** 2022-01-25

**Authors:** Catherine Olesch, David Brunn, Öznur Aktay-Cetin, Evelyn Sirait-Fischer, Soni Savai Pullamsetti, Friedrich Grimminger, Werner Seeger, Bernhard Brüne, Andreas Weigert, Rajkumar Savai

**Affiliations:** ^1^ Institute of Biochemistry I, Goethe-University Frankfurt, Frankfurt, Germany; ^2^ Max Planck Institute for Heart and Lung Research, Member of the German Center for Lung Research (DZL), Member of the Cardio-Pulmonary Institute (CPI), Bad Nauheim, Germany; ^3^ Institute for Lung Health (ILH), Justus Liebig University, Giessen, Germany; ^4^ Department of Internal Medicine, Justus Liebig University Giessen, Member of the DZL, Member of CPI, Giessen, Germany; ^5^ Frankfurt Cancer Institute (FCI), Goethe University, Frankfurt am Main, Germany; ^6^ German Cancer Consortium (DKTK), Frankfurt, Germany

**Keywords:** lung cancer, tumor microenvironment, lung tumor heterogeneity, multispectral flow cytometry, hierarchical clustering

## Abstract

The lung tumor microenvironment plays a critical role in the tumorigenesis and metastasis of lung cancer, resulting from the crosstalk between cancer cells and microenvironmental cells. Therefore, comprehensive identification and characterization of cell populations in the complex lung structure is crucial for development of novel targeted anti-cancer therapies. Here, a hierarchical clustering approach with multispectral flow cytometry was established to delineate the cellular landscape of murine lungs under steady-state and cancer conditions. Fluorochromes were used multiple times to be able to measure 24 cell surface markers with only 13 detectors, yielding a broad picture for whole-lung phenotyping. Primary and metastatic murine lung tumor models were included to detect major cell populations in the lung, and to identify alterations to the distribution patterns in these models. In the primary tumor models, major altered populations included CD324^+^ epithelial cells, alveolar macrophages, dendritic cells, and blood and lymph endothelial cells. The number of fibroblasts, vascular smooth muscle cells, monocytes (Ly6C^+^ and Ly6C^–^) and neutrophils were elevated in metastatic models of lung cancer. Thus, the proposed clustering approach is a promising method to resolve cell populations from complex organs in detail even with basic flow cytometers.

## Introduction

Lung cancer continues to be a leading cause of cancer-associated mortality worldwide, accounting for about 1.8 million deaths in 2020 (1). Current therapies aim to target the components of the lung tumor microenvironment (TME) to interfere with cancer progression. Since the TME plays a crucial role in tumor progression and metastasis, precise identification of the cell populations in the TME can contribute to the development of effective novel targeted therapies (2). The crosstalk between cancer cells and stromal cells sustains the oncogenic microenvironment, thus the quantity and distribution of cells in the TME are likely associated with the pathogenesis of lung cancer. For instance, tumor-associated macrophages (TAMs) are among the most abundant cell types in the lung TME, and TAM infiltration is reportedly correlated to the stage and metastasis potential of lung cancer (3, 4). Since the involvement of microenvironmental cells in disease progression is multifaceted, profiling the TME is critical to elucidate cellular activity, interactions and functions to establish more specific targeted approaches to inhibit lung tumorigenesis. There are currently various techniques available to unravel the cellular composition of the TME such as basic immunohistochemistry, multiplex staining, flow cytometry, mass cytometry, transcriptomic and bioinformatics approaches.

Polychromatic flow cytometry has been extensively used for characterization of cell populations in lymphoid and non-lymphoid tissues of different species (5–7). Therefore, the focus is usually on immune cells based on the relative ease of isolation from tissues and the availability of established high-quality antibodies targeting immune cell surface markers. When paired with the use of a counting standard, flow cytometry can yield reliable information not only on the activation status and absolute number of immune cells in tissues. Current multispectral flow cytometry approaches usually do not integrate the analysis of different types of tissues from an organ, i.e. combining the analysis of immune cells with other stromal and/or epithelial cells from distinct tissues of a particular organ, largely due to differences in protocols for creating single cell suspensions, and the limited number of available fluorescence channels. However, such integrated analyses may be required to elucidate complex biological phenotypes, especially since the amount of available tissue from mouse models is relatively limited. For instance, a reduced immune infiltrate can result from altered chemokine production or perturbed formation of the vasculature which can reduce immune cell infiltration (8). Understanding the relative contribution of immune cells in general and immune cell subsets, in particular, the tissue architecture is key to clarifying the relative contribution to an observed phenotype. We are not aware of available flow cytometry panels for detailed analysis of tissue complexity beyond the number and type of immune cells.

In-depth characterization of immune cell phenotypes including activation states *via* flow cytometry is limited, similar to analysis of tissue complexity. Hence, mass cytometry was developed to overcome the limitation of available parameters for single cell analysis by flow cytometry. Mass cytometry currently allows for high-throughput screening of up to 50 parameters of single cells from large numbers of cells of an experimental sample, with similar accuracy and reproducibility as conventional flow cytometry (9–11). The advantages of mass cytometry as compared to flow cytometry include the lack of spill-over and therefore a relative ease of panel design, as well as the absence of autofluorescence, which can influence the reliability of flow cytometry (12). However, there are several disadvantages to mass cytometry including the destruction of cells for analysis, making it impossible to use analyzed cells in downstream assays, lower throughput, and signal loss (only ~ 25% of all cells and 0.05% of ions are recorded), which impacts the discovery of rare cell subsets in small tissue samples, and low efficacy of filtering out debris and doublets. In addition, the procurement and running costs of a mass cytometry are unaffordable for many laboratories around the world. Due to these limitations, there is still a need to develop approaches to increase the dimensionality of conventional flow cytometry.

Here, we describe a procedure to isolate and analyze not only immune cells but also a number of stromal and epithelial cell populations from mouse lungs under steady-state conditions and in response to metastatic cancer. A hierarchical clustering method that allows utilization of a number of fluorochromes coupled to more than one antibody was employed with 13 detectors. Combined with physical parameters and a live/dead dye, this flow cytometry panel can analyze 27 parameters in murine tissue to simultaneously enumerate major leukocyte populations, as well as cells of the vascular system, fibroblasts, and epithelial cells. Combined with next-generation flow cytometers, this approach allows greater dimensionality than mass cytometry.

## Results

### FACS Analysis of Lung Tumor Heterogeneity

To determine whether flow cytometry can detect major cell subsets in control lungs, a multispectral antibody panel (**Table 1**) was designed that employed hierarchical clustering and individual fluorochromes that will allow more than one marker for one particular cell subset that was previously separated by other broad markers of cell subsets (**Supplementary Figure S1**). For instance, fluorescein isothiocyanate (FITC) or AlexaFluor 488, measured in the same channel, were assigned to antibodies recognizing melanoma cell adhesion molecule (MCAM or CD146), sialic acid binding Ig-like lectin H (Siglec-H), and glucocorticoid-induced tumor necrosis factor receptor (GITR). CD146 is expressed by blood endothelial cells (BECs) and vascular smooth muscle cells (VSMCs), which can be separated by differences in the expression patterns of platelet endothelial cell adhesion molecule (PECAM-1 or CD31), which is highly expressed on the surfaces of endothelial cells, while Siglec-H is expressed exclusively by plasmacytoid DCs (pDCs) and GITR is expressed at particularly high levels by regulatory T cells (Tregs). Both pDCs and Tregs, can be separated from ECs and VSMCs by staining for CD45 expression and pDCs can be distinguished from Tregs by CD3 expression in the latter. Allophycocyanin (APC) was assigned to epithelial cadherin (E-cadherin or CD324), major histocompatibility complex II (MHC II) and the γδ T cell receptor (γδ TCR). Epithelial cells express CD324 but not MHC II or the γδ TCR. MHC II is expressed on the surface of a variety of cells, but not on mouse T cells, which are differentiated by CD3 expression. Thus, MHC II is not co-expressed with the γδ TCR or CD324. Nevertheless, in some instances, different markers were intentionally labeled with the same fluorochrome, as in the case of the Fc-gamma receptor 1 (FcγRI or CD64) and Mer tyrosine kinase (MerTK), which are both highly expressed on the macrophage surface. Here, co-expression was used to separate cell populations. Markers that are common to different cell populations, such as lymphocyte antigen 6 complex locus C (Ly6C), which is expressed on a subset of monocytes, granulocytes, lymphocytes, fibroblasts and vascular endothelial cells or that separate a larger cell subset from other cells such as CD45 (all immune cells) or CD3 (all T cells), were assigned to a fluorochrome that was only used once (**Table 1**).

**Table 1:** Multispectral antibody panel.

Panel	Antigen	Label	Cell type	Dilution	RRID
Lung endothelium	CD31	PE-Cy7	BECs, LECs	1:5000	AB_2716949
	CD90	PE	LECs	1:50	AB_2659874
	CD146	AF488	BECs	1:100	AB_11153320
	Ly6C	PerCP-Cy5.5	BECs	1:100	AB_1727558
Lung epithelium and stroma	CD90	PE	Fibroblasts	1:50	AB_2659874
	CD117	APC-eFluor780	Epithelial cells	1:200	AB_1582226
	CD140	PE	Fibroblasts	1:50	AB_2737787
	CD146	AF488	VSMCs	1:100	AB_11153320
	CD324	AF647	Epithelial cells	1:100	AB_2563955
	CD326	BV711	Epithelial cells	1:100	AB_2738022
Lung myeloid	CD11b	BV605	Monocytes Eosinophils	1:200	AB_2737951
			Neutrophils		
	CD11c	BV711	AMs	1:50	AB_2734778
	CD24	PE-CF594	cDCs	1:333	AB_11151917
	CD44	AF700	AMs	1:50	AB_1727480
	CD45	VioBlue	All leukocytes	1:50	AB_2659925
	CD64	PE-Cy7	IMs	1:50	AB_2563904
	CD117	APC-eFluor780	DCs, Mast cells	1:200	AB_1582226
	Ly6C	PerCP-Cy5.5	Monocytes	1:100	AB_1727558
	Ly6G	APC-Cy7	Neutrophils	1:66	AB_10640819
	MerTK	PE-Cy7	IMs	1:50	AB_2617037
	MHCII	APC	cDCs, IMs	1:200	AB_313329
	SiglecF	PE	Eosinophils	1:50	AB_394341
Lung lymphoid	CD24	PE-CF594	B cells	1:333	AB_11151917
	CD3	APC-Cy7	T cells	1:50	AB_1727461
	CD4	BV711	CD4+ T cells	1:100	AB_2737973
	CD8a	BV650	CD8+ T cells	1:200	AB_2563056
	CD90	PE	Lymphocytes	1:50	AB_2659874
	GITR	FITC	Tregs	1:100	AB_1089125
	NK1.1	BV510	NK cells	1:100	AB_2738002
	SiglecH	FITC	pDCs	1:100	AB_1227761
	γδ TCR	APC	γδ T cells	1:33	AB_1731813

Antibodies, and respective fluorochromes, that were used for identification of lung cell populations by FACS analysis. AMs, Alveolar macrophages; BECs, Blood endothelial cells; cDCs, Conventional dendritic cells; IMs, Interstitial macrophages; LECs, Lymphatic endothelial cells; NK, Natural killer; pDCs, Plasmacytoid dendritic cells; Tregs, Regulatory T cells; VSMCs, Vascular smooth muscle cells.

**Figure 1:**
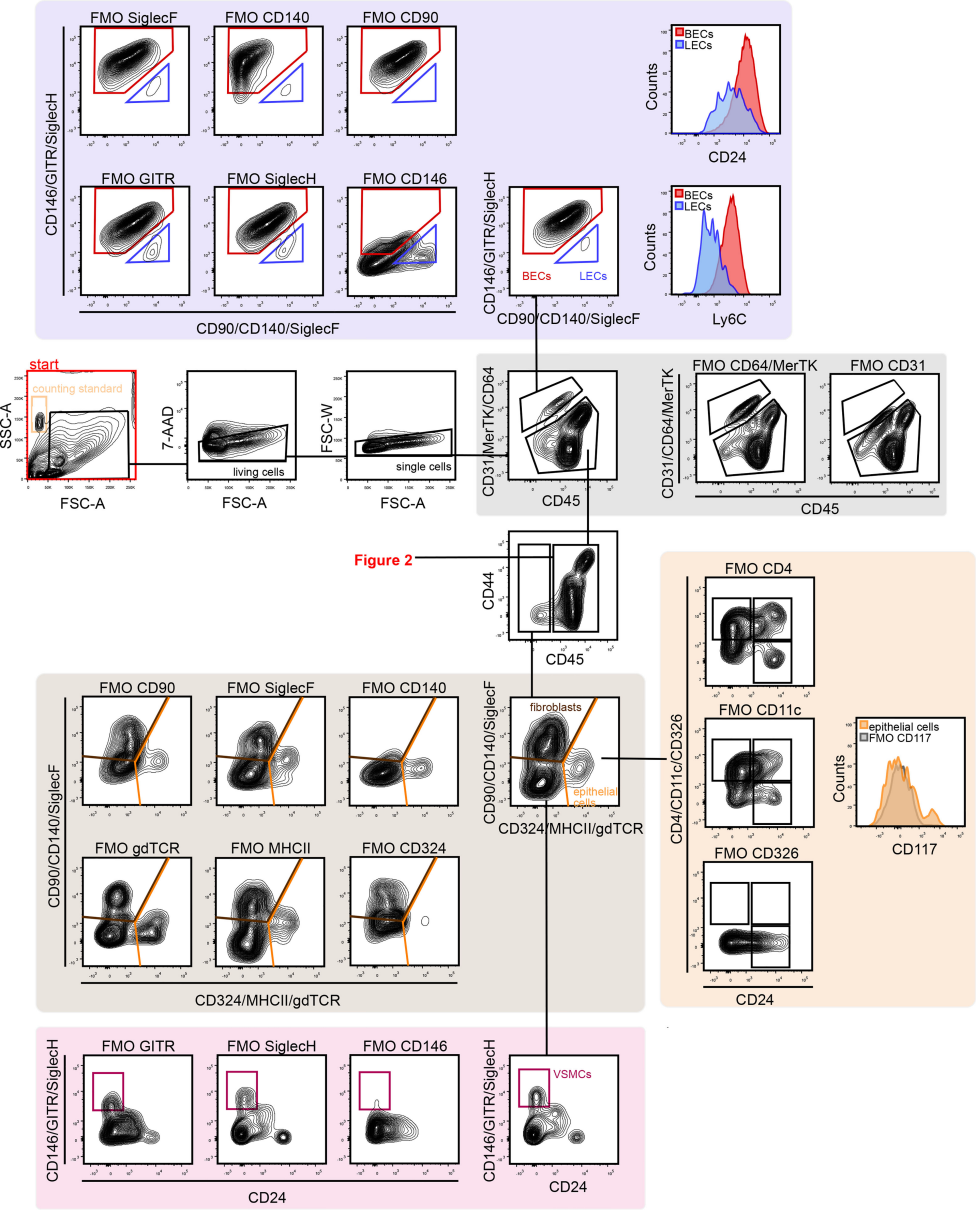
Gating strategy for lung vascular, epithelial and stromal cells. First, FSC, SSC, and 7-AAD gating was conducted to remove debris, dead cells, and cell clumps. Next, vascular cells were separated from other lung cells by CD31 and CD45 gating. CD31^+^ cells are vascular endothelial cells, which can be separated into BECs (CD146^+^ Ly6C^+^) and LECs (CD90^+^ CD146^-^). CD45^+^ cells are immune cells (see **Figure 2**). The CD31^–^/CD45^–^ population included fibroblasts (CD140^+^/CD324^–^) and epithelial cells (CD324^+^/CD140^–^). The CD324^–^/CD140^–^ population included CD146^+^ VSMCs. 7-AAD, 7-aminoactinomycin D; BECs, blood endothelial cells; FMO, fluorescent minus one; FSC-A, forward scatter area; FSC-W, forward scatter width; LECs, lymphatic endothelial cells; SSC, side scatter; VSMCs, vascular smooth muscle cells.

**Figure 2:**
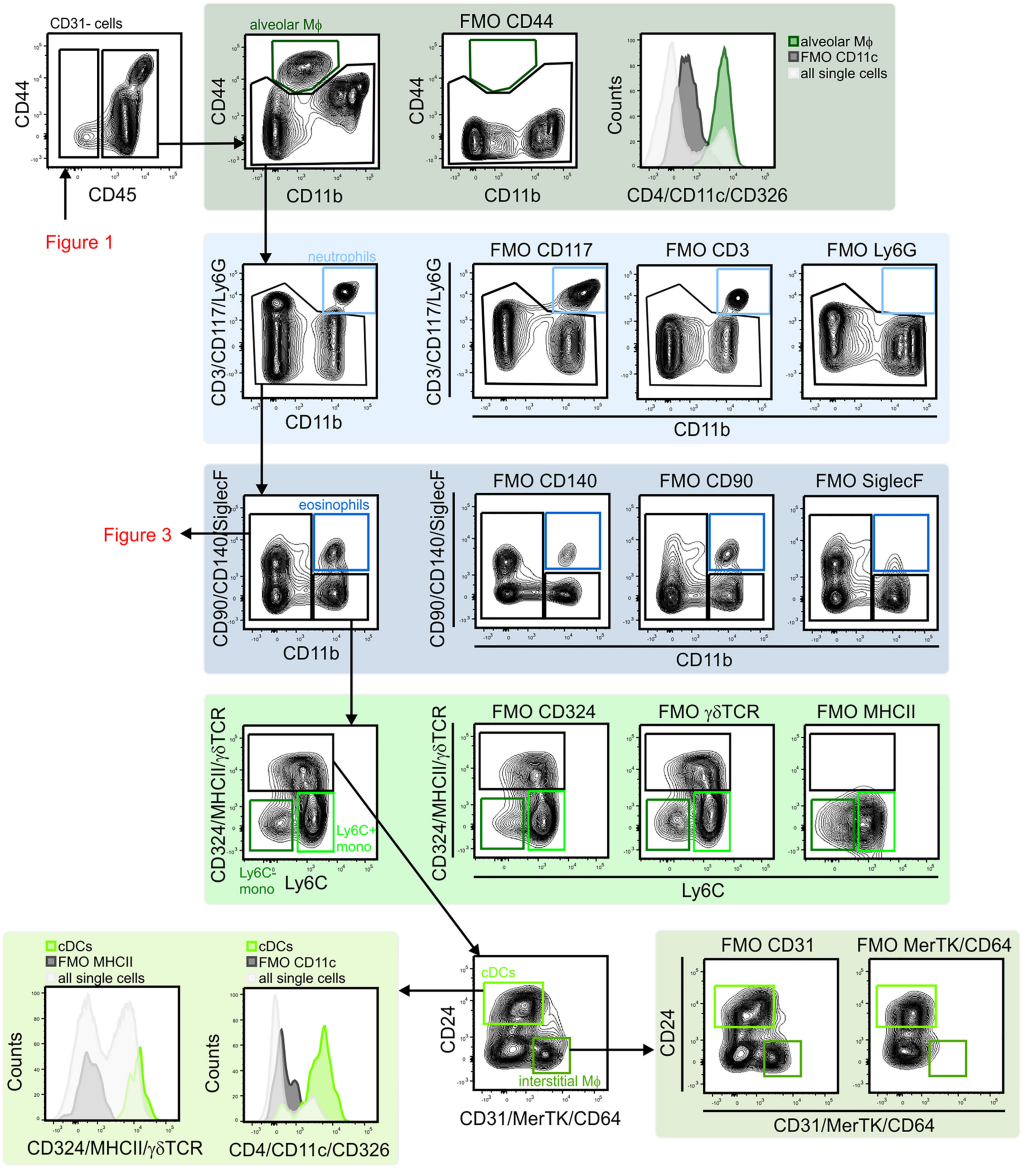
Gating strategy for lung myeloid cells. The CD45^+^ population included alveolar macrophages (CD44^+^/CD11b^–^/CD11c^+^) were identified. CD11b expression was used to distinguish myeloid from lymphoid cells. The CD11b^+^ population included neutrophils (CD11b^+^/Ly6G^+^), eosinophils (CD11b^+^/SiglecF^+^), monocytes (MHCII^–^/Ly6C^+^ and MHCII^–^/Ly6C^–^), cDCs (MHCII^+^/CD24^+^) and interstitial macrophages (MHCII^+^/CD24^–^/CD64/MerTK^+^) were identified. cDCs, conventional dendritic cells; FMO, fluorescent minus one; Mono, monocytes; MФ, macrophage.

**Figure 3:**
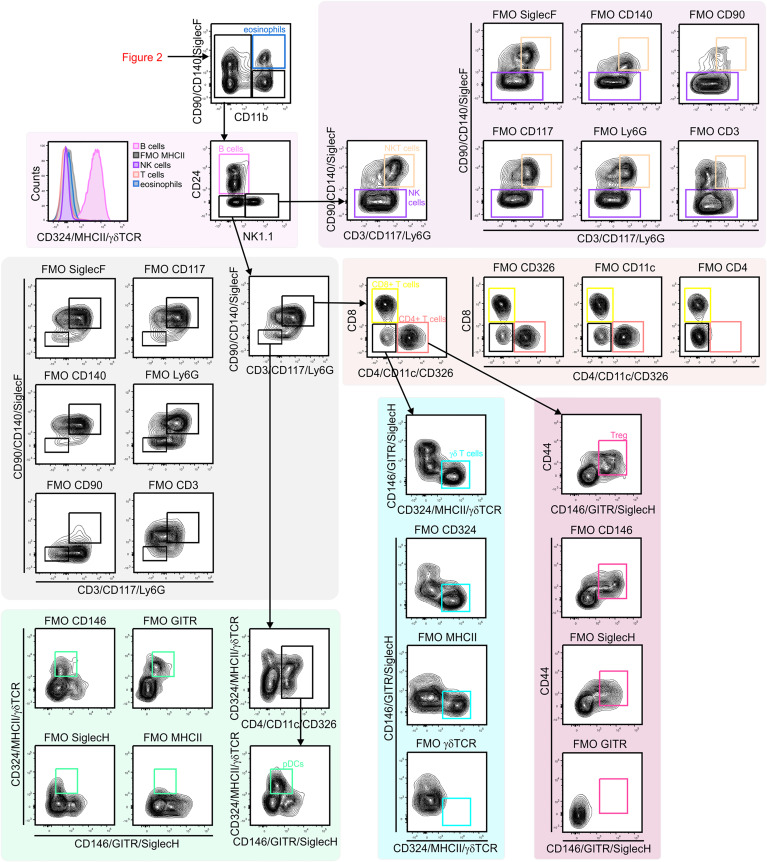
Gating strategy for lung lymphoid cells. CD11b^–^ lymphocytes were first separated based on CD24 and NK1.1 expression to identify B cells (CD24^+^/MHCII^+^) and NK1.1 single positive cell (CD24^–^/NK1.1^+^). NK1.1^+^ population contained CD3^–^ NK cells and CD3^+^/CD90^+^ NKT cells. CD24^–^/NK1.1^–^ population included CD3^+^ T cell subsets (CD4^+^ T cells, CD8^+^ T cells, Treg cells (CD4^+^/GITR^–^) and γδ T cells (γδ TCR^+^/CD4^–^/CD8^–^). Within CD3^–^ cells pDCs (CD11c^+^/MHCII^+^ SiglecH^+^) were identified. NK, natural killer; NKT cell, natural killer T cell; pDC, plasmacytoid dendritic cell.

The use of a single fluorochrome for multiple markers is hampered by a number of practical difficulties, including differences in marker expression levels, leading to potential off-scale expression. For instance, potent antibodies are available against CD31, which is highly expressed on endothelial cells. CD31 was labeled with PE-Cy7, as were potential macrophage markers during panel testing. Adjusting photomultiplier tube voltages to accommodate CD31 expression led to vanishing of the background expression of single macrophage markers, which was the reason for using the same fluorochrome for two macrophage markers simultaneously. Another difficulty is the use of compensation beads that non-specifically recognize antibodies. Differences in the affinity of antibodies labeled with the same fluorochrome will lead to difficulties during compensation due to issues with selecting a proper positive population. Therefore, antibodies labeled with the same fluorochrome must have comparable affinities to the compensation beads. Finally, florescence minus one (FMO) controls were essential to validate the approach and to ensure proper identification of immune cell populations (**Figures 1**–**3**).

The technique outlined above was used to identify 20 discrete cell populations in healthy mouse lungs. First, living single cells were identified by removing debris *via* forward side (FSC)-A versus side scatter (SSC)-A gating, followed by removal of dead cells positive for 7-aminoactinomycin D (7-AAD) cells and by gating out doublets *via* FSC-A versus FSC-W gating (**Figure 1**). Next, the vascular compartment cells were separated from other cell types by comparing CD31 and CD45, since immune cells also express CD31, albeit at considerably lower levels than endothelial cells. FMO controls clearly demonstrated the presence of a CD31^+^/CD45^–^ population. The vascular compartment of the lung contains vascular and lymphatic ECs. Vascular ECs, according to single cell RNA-seq databases such as the Immgen database (13), express high levels of CD146, Ly6C and CD24, in contrast to lymphatic endothelial cells, which express high levels of CD90. Corresponding populations were identified in the vascular fraction, with the majority of this cell fraction consisting of vascular endothelium, in agreement with previously published single cell RNA-seq data (14). Next, CD45^–^ cells were separated from CD45^+^ for further analysis (**Figure 1**). CD45^–^ cells were first divided into CD140^+^ fibroblasts, CD326^+^ epithelial cells and CD140^–^/CD326^–^ cells. Within the latter, CD146^+^/CD24^–^ cells were designated as VSMCs. There was also a distinct CD24^+^ subset in the CD146^–^ stromal compartment, although this population was not further characterized (**Figure 1**). In the epithelial fraction, a number of different subpopulations based on the expression patterns of epithelial cell adhesion molecule (EpCAM or CD326), CD24 and tyrosine-protein kinase KIT (CD117), were observed that may correspond to type I versus type II alveolar and bronchial epithelial cells as well as associated progenitor cells, although these cells were not completely characterized.

Next, subsets of CD45^+^ immune cells were identified (**Figures 2** and **3**). Alveolar macrophages were identified by high expression of the hyaluronan receptor CD44, low expression of CD11b, and high expression of CD11c (**Figure 2**). It was essential to remove these highly autofluorescent cells first, to avoid the production of autofluorescence artifacts in order to identify granulocyte subsets. Neutrophils were positive for CD11b and lymphocyte antigen 6 complex locus G6D^+^ (Ly6G^+^), while eosinophils were positive for CD11b and SiglecF (**Figure 2**). The remaining myeloid cells in the CD11b^+^/Ly6G^–^/SiglecF^–^ compartment were also analyzed. Ly6C^+^ cells which did not express MHCII were designated as Ly6C^+^ monocytes, and Ly6C^–^/MHCII^–^ cells as Ly6C^–^ monocytes. The MHCII^+^ population was further subdivided into MHCII^high^/CD24^+^/CD64/MerTK^–^ conventional dendritic cells (cDCs) and CD24^–^/CD64/MerTK^+^ interstitial macrophages (**Figure 2**). The remaining MHCII^+^ cells and potential myeloid cells within the Ly6C^–^ monocyte populations (due to the lack of a positive marker for this population) may contain basophils or CD11b^+^ natural killer (NK) cells. The CD11b negative compartment mainly consisted of lymphocytes, but no pDCs (**Figure 3**). CD11b^–^ cells were first separated into CD24^+^ and NK1.1^+^ subsets as well as CD24^–^/NK1.1^–^ cells. CD24^+^ cells were MHCII^+^ B cells and the NK1.1^+^ cell subset consisted mainly of CD3^+^/CD90^+^ natural killer T (NKT) cells and CD3^–^/CD90^–^ NK cells (**Figure 3**). The CD24^–^/NK1.1^–^ subset contained CD3^+^/CD90^+^ T cells, which were further separated into CD4^+^, CD8^+^ and CD4^–^/CD8^–^ T cell subsets. The CD4^+^ T cell population also included GITR^+^/CD44^+^ regulatory T cells, while the CD4^–^/CD8^–^ T cell population contained a large proportion of γδ T cells (**Figure 3**). The CD11b^–^ cell population likely contained a number of other cell populations. Of these, cells expressing CD4 and/or CD11c, as well as intermediate levels of MHCII and Siglec-H, were therefore designated as pDCs (**Figure 3**). Thus, through the proposed antibody panel and the hierarchical approach including FMO controls, we were able to identified 20 distinct lung cell populations with relative ease, while further populations, such as the epithelial cell compartment, also emerged.

### Increased Interstitial Macrophages, DCs and Tregs in Different Lung Cancer Models

**Figure 4:**
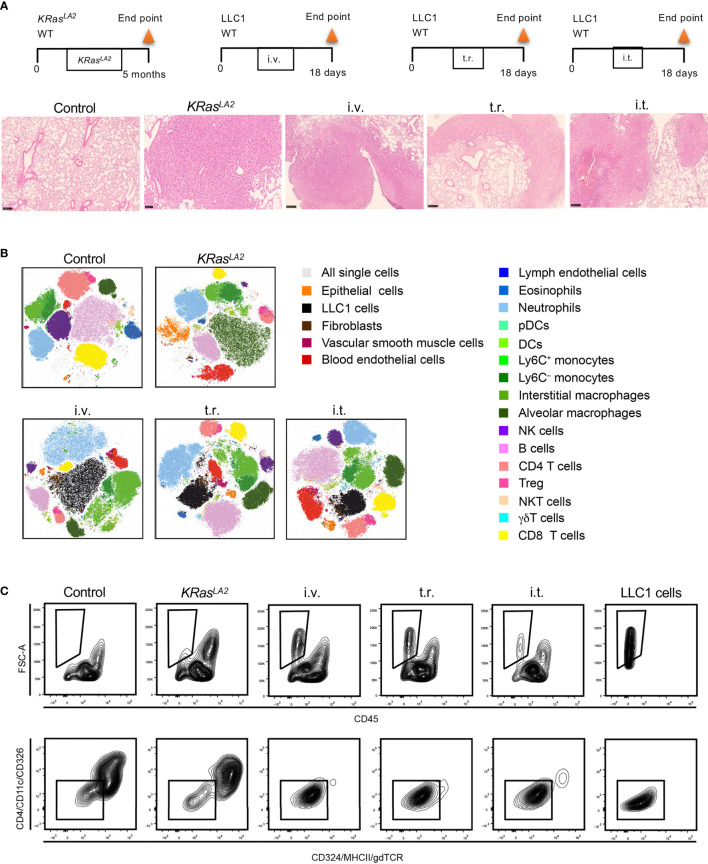
Cell composition in different murine lung cancer models. **(A)** Schematic representation of primary and metastatic lung tumor models in mice (upper panel). Histological analysis of lung tumors from given models (lower panel). H&E staining of lung tissue sections to reveal tumor formation (scale bar=250µm). **(B)** t-SNE plots show distribution of pulmonary cell compositions of different lung cancer models. **(C)** Strategies to identify LLC1 cells, which lack conventional epithelial markers in tumor models are depicted. Control means healthy lung. i.t., intratracheal injection; i.v., intravenous injection; t.r., tumor relapse model; WT, wild type; H&E, hematoxylin and eosin; DC, dendritic cells; pDCs, plasmacytoid dendritic cells; NK, natural killer; NKT cells, natural killer T cells, Treg, regulatory T cells; t-SNE, T-distributed stochastic neighbor embedding.

Next, the usefulness of the panel to characterize alterations in lung cell composition in different pathological settings was explored using four different but widely used lung cancer models. Therefore, we took account of four primary and metastatic lung tumor models as follows: (i) transgenic *KRas^LA4^* model, (ii) intratracheal instillation model, (iii) intravenous injection model and (iv) tumor relapse model (**Figure 4A**).

**Figure 5:**
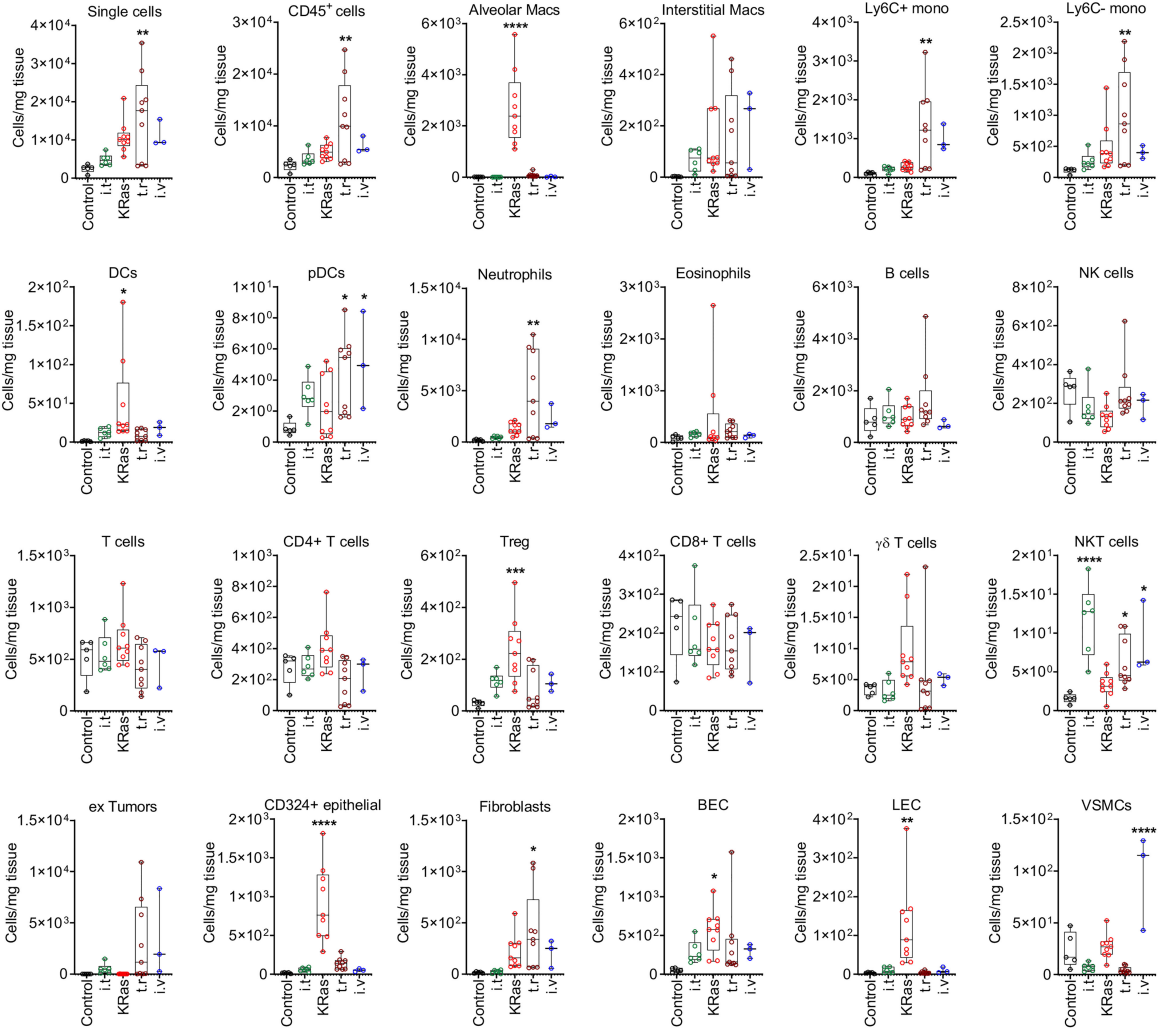
Quantitative distribution of pulmonary cells in different lung tumor models. Cell numbers (per 1 mg of tissue) determined by FACS analysis in healthy control lung and cancerous lungs from four different models are shown. Control means healthy lung. i.t., intratracheal injection; i.v., intravenous injection; t.r., tumor relapse model; Macs, macrophages; Mono, monocytes; DCs, dendritic cells; pDCs, plasmacytoid dendritic cells; NK cells, natural killer cells; NKT cells, natural killer T cells; Treg, regulatory T cells; BECs, blood endothelial cells; LEC, lymphatic endothelial cells; VSMCs, vascular smooth muscle cells. Numbers of mice by group: control n=5, for i.t. model n=6, for *KRas* model n=9, for tumor relapse model n=9, for i.v. model n=3. *P < 0.05, **P < 0.01, ***P < 0.001, ****P < 0.0001 compared with control.

Flow cytometry standard files were concatenated and subjected to T-distributed stochastic neighbor embedding (t-SNE) dimensionality reduction to visualize population changes upon the development/injection of primary and metastatic lung cancer (**Figure 4B**). These data as well as quantitative data calculated using a counting standard (**Figure 5** and **Supplementary Figure S2**) revealed similarities but also clear discrepancies among the four models. Importantly, a new population of FSC high cells negative for CD45, CD324 and CD326, as well as other major lineage markers such as CD90 and CD31, (not shown), was observed in all models containing LLC1 cells, including the metastasis model. A comparison with LLC1 cells from cell culture revealed that these cells indeed were LLC1 cells (**Figure 4C**).

The proportions of these cells were significantly elevated as compared to control lungs with the transgenic *KRas^LA2^*, intravenous injection, and tumor relapse models, but not the intratracheal instillation model (**Figure 4B**). In contrast, the *KRas^LA2^* model showed a significant increase in the number of CD324^+^ epithelial cells. Additionally, the populations of alveolar macrophages, DCs, BECs and LECs were significantly upregulated in only the *KRas^LA2^* model as compared to control lungs. With the exception of intratracheal instillation tumors, the numbers of Tregs were significantly elevated in three of the tested models. Besides Ly6C^+^ and Ly6C^–^ monocytes, pDCs and neutrophils, the number of fibroblasts was also significantly enhanced in the tumor relapse model. Interestingly, the number of pDCs tended to increase in the tumor relapse and intravenous injection models. NKT cell numbers were significantly augmented in all of the tested primary and metastatic models with the exception of the transgenic *KRas^LA2^* model. The only significant increase in the number of VSMCs was observed with the intravenous injection lung tumor model (**Figure 5**).

## Discussion

The lung tumor microenvironment is a complex structure and it plays an active role in the later stages of tumorigenesis and metastasis. The major contributor of this malignant transformation is the dynamic crosstalk between cancer cells and stromal cells, in which the abundance and localization of cells are correlated to disease progression (3, 4). The lung architecture is a habitat for around 40 different cell types including residential cells of the respiratory tract and pulmonary arteries (15). Therefore, precise profiling of lung cellular composition in health and disease is crucial for the development of efficient targeted therapies.

In the present work, a multispectral FACS panel was established using hierarchical clustering to identify distinct cell populations of healthy and tumor lungs. The proposed multispectral panel uses fluorochromes that coupled to more than one antibody to examine a total of 24 parameters with 13 detectors. Testing with four different lung tumor models we found that cell populations differed between the primary and metastatic lung cancer models. For example, the numbers of CD324^+^ epithelial cells, alveolar macrophages, BECs and LECs were significantly increased in primary tumors of the transgenic *KRas^LA2^* model. Moreover, the proportion of cDCs was also significantly increased. However, quantities of pDCs were found in metastatic models only. A study with human primary lung carcinoma biopsy samples revealed high numbers of CD11c^+^ DCs but not pDCs (16), which supports our findings regarding DC subtypes. Elevated Treg numbers in primary tumors, especially in the *KRas^LA2^* model, was not surprising since high Treg levels were previously detected in the *KRas^G12D^* model (17). Additionally, a previous study reported that the number of Tregs was relatively high in primary lung adenocarcinoma (18). In the current study, stromal cells were abundant in the metastatic tumor models. For instance, fibroblasts were only significantly elevated in the tumor relapse model, whereas VSMC numbers were significantly increased only in the intravenous injection model. As a possible explanation for this result, metastatic cells localize in the lungs (19). A previous *in vivo* study showed that activation of cancer-associated fibroblasts contributes to a pre-metastatic environment in the lung and enhances metastasis of salivary adenoid cystic carcinoma to the lungs (20). Moreover, it was also reported that lung metastasis of breast cancer increased the number of fibroblasts in the lungs during colonization (21). These examples help to explain the high quantity of fibroblasts in the metastasis models. There were notable increases in the numbers of monocytes (Ly6C^+^ and Ly6C^–^) and neutrophils in the metastasis models. A recent study showed that the number of Ym1^+^/Ly6C^hi^ monocytes was increased in the peripheral blood of mice with early stages of lung metastasis, suggesting that the proportion of monocytes can be used as a biomarker of metastasis (22). Another study reported that injection of LLC1 cells to mice with *Ifnar1* promoted the development of lung metastasis together with the accumulation of neutrophils in the lung, indicating the involvement of neutrophils in metastasis (23). Additionally, as compared to controls, primary lung tumors had greater proportions of CD45^+^ cells as determined with the metastatic tumor relapse model. Interestingly, the proportion of type 1 NKT cells expressing NK1.1 was increased in three of four tested models but not in the *KRas^LA2^* model. The anti-metastatic function of NKT cells might have been associated with the elevated cell numbers in the metastatic models. For instance, type 1 NKT cells had an anti-metastatic effect on the mouse liver and lungs (24). Moreover, type 1 NKT cells, have also been detected in primary human lung tumor tissue (25). However, in mouse strains expressing NK1.1, NK1.1^+^ T cells, including type I NKT cells, as well as activated CD8^+^ T cells and γδ T cells were activated (26, 27). Thus, further studies are needed to clearly delineate the function of these cells in models of tumor metastasis.

Analysis of other T cell subpopulations (CD4^+^, CD8^+^ and γδ T cells), interstitial macrophages, eosinophils, B cells and NK cells revealed no significant change in primary versus metastatic tumors. A lack of alteration in T cell subsets is supported by previous transcriptomic analysis of stromal cells of *KRas*-driven mouse tumors, which found no change in the number of CD4^+^ and CD8^+^ T cell numbers between tumor and control tissue of the lungs (28).

Quantification of cell populations in the lung TME should be precise because single cell subsets in lung tumors may, to some degree, be crucial for the development of targeted therapies. Flow cytometry is the method of choice to reliably quantify cells within tissues once a suitable method to create a single cell suspension is available. Methods such as immunohistochemistry (IHC) and immunofluorescence analyses require staining of large numbers of serial sections to achieve similar accuracy of quantification. However, spatial information of cell distribution throughout the tumor is retained by these methods, but is lost during the generation of single cell for flow cytometry.

Transcriptomic approaches have been employed for more in-depth identification and characterization of cell populations (29, 30), but lack of the accurate quantification and low cell input number may result in overlooking small, but important populations. Thus, each technique to interrogate tissue heterogeneity has both advantages and limitations to sample throughput, sample preparation, analysis, time and cost, and need to be performed in combination to produce meaningful results.

With current technology, up to 50 parameters can theoretically be analyzed by flow cytometry, although a sufficiently diverse palette of fluorochromes is still lacking. Our strategy may serve to overcome these limitations and allow laboratories with smaller budgets to perform high-dimensional analyses as well. The panel described herein can of course be improved and extended. For instance, epithelial cells can be further segregated based on the expression patterns of other markers, such as EpCAM (type II^+^, type I^–^) (31). It is also possible to develop new markers of stromal cells and epithelial cells by labeling with fluorochromes, similar to those used for the immune cell compartment such as BV650 or BV510 for multicolor flow cytometry. For compensation of channels in which fluorochromes were used more than once, antibodies coupled to fluorochromes measured in one channel were pooled. When these antibodies were conjugated with the same fluorochrome, preferentially by the same provider, single signal peaks were detected during acquiring compensation controls. Using compensation beads was instrumental for this approach. When two peaks were observed (only applied to the APC-eFluor780/APC-Cy7 channel), these were close in intensity. However, upon visual inspection, focusing compensation on the peak with the higher intensity proved to be superior. To avoid issues with tandem degradation, antibodies were strictly kept at 4°C and light exposure was reduced to the absolute minimum. Moreover, when changing antibody lot numbers, compensation matrices were adjusted by acquiring compensation controls for the changed lots individually in pre-existing compensation setups. This was made possible by daily calibration of the instrument using Cytometer Setup and Tracking. In addition, advanced bioinformatics approaches may be needed to analyze data generated by the proposed technique at sufficient depth and speed. Such developments are, however, currently underway as suggested by a report of a machine learning approach to assess flow cytometry data to characterize the distribution of immune inflammatory cells in the lungs of a bleomycin mouse model (32).

The proposed panel is not only useful or screening of cancers but also other pulmonary diseases that progress *via* all resident cells and recruited immune cells contribute to the progression of pulmonary diseases, such as lung fibrosis and pulmonary hypertension (33). It can also be envisioned for use in other tissues as well. Indeed, a reduced version has already been applied for screening of skin specimens (34). Additionally, the panel can be adapted for use in human lung cancer, i.e. due to similarities between human and mouse macrophages (35), although adjustments will be needed to account for species differences, such as MHCII expression among subsets of human T cells.

In conclusion, a hierarchical method was applied to assess alterations in lung cell populations among the four different murine lung tumor models. The cellular distribution of microenvironmental cells also varied between the primary and metastatic models of lung cancer, which can yield additional information regarding tumor progression. Picturing the cellular landscape of the lung in states of both health and disease is critical to improve the effectiveness of targeted therapies. Therefore, future studies are warranted for the design of precise panels for the identification of cells in complex organs and systems will guide us to decode the cellular crosstalk in diseases, especially in cancer.

## Materials and Methods

### Cell Culture

Murine Lewis lung carcinoma (LLC1) cells were obtained from American Type Culture Collection (ATCC; CRL-1642, Manassas, VA, USA) and cultured in Roswell Park Memorial Institute 1640 (RPMI; Gibco, 11879-020, Gibco, Carlsbad, CA, USA), supplemented with 10% fetal calf serum (FCS) and 1 U/ml penicillin-streptomycin under an atmosphere of 5% CO_2_/95% air at 37°C and 5% CO_2_ in an incubator (Heracell 240i, Thermo Fisher Scientific, Waltham, MA, USA) for 2-3 days. and medium was replaced by fresh medium regularly every two to three days. At 70% - 80% confluence, the cells were washed with 1X phosphate buffered saline pH 7.4 (PBS; Gibco, 10010056) and then treated with 0.05% Trypsin-EDTA (Gibco, 25300-054) at 37°C.

### Animal Experiments

Mice were kept under specific pathogen-free conditions in individual ventilated cages (IVC). C57BL/6 mice were purchased from Charles River Laboratories (Sulzfeld, Germany). *KRas^LA2^* mice were purchased from Jackson Laboratory (Sulzfeld, Germany) and bred in-house with C57BL/6 mice. In this study, four different lung tumor models were used: (i) *KRas^LA2^* model: the *KRas^LA2^* mutation leads to an amino acid exchange of glycine to aspartic acid at codon 12. Mice homozygous for the *KRas^LA2^* mutation die during embryogenesis, whereas heterozygous mice have no congenital abnormalities. *KRas^LA2^* mice produce active *KRas*, which leads to lung tumors. *KRas^LA2^* mouse lungs were harvested at the end point (26 weeks) for single cell suspension preparation and histology analysis; (ii) intratracheal (i.t.) injection model: 1x10^6^ LLC1 cells were resuspended in a final volume of 100 µl of 0.9% NaCl and injected into the trachea of C57BL/6 mice. On day 20, the lungs were harvested for FACS and histological analysis, (iii) intravenous (i.v.) tumor model: LLC1 cells (1x10^6^) were injected into the tail vein. On day 18, the mice were sacrificed, and the lungs were harvested for FACS and histological analysis. (iv) tumor relapse model: primary tumor growth was initiated by subcutaneous injection of LLC1 cells (1x10^6^). Tumor resection of anesthetized mice was performed on day 8. Lung metastasis was observed for 30 days after tumor resection prior to harvesting the lungs for further examinations.

### Generation of Single Cell Suspensions

To prepare lung tumors for flow cytometric analysis, the extracted lungs were weighed, placed in 35 mm × 10 mm petri dishes (Greiner Bio-One, Kremsmünster, Austria), and cut into small cubes (< 1 mm^3^) with a scalpel. Single cell suspensions were created using the Tumor and Lamina Propria dissociation kit (Miltenyi Biotec, Bergisch Gladbach, Germany) and the GentleMACS isolator (Miltenyi Biotec) according to manufacturer’s protocol. The resulting pellet was washed with PBS and passed through a 40-mm cell strainer (BD Biosciences, San Jose, CA, USA) before FACS analysis of single cell suspensions.

### Flow Cytometry

Samples were acquired with a LSRII/Fortessa flow cytometer (BD Biosciences) and data analysis was performed using FlowJo software v10 (Tree Star, Inc., Ashland, OR, USA). All antibodies and secondary reagents were titrated to determine optimal concentrations. The excitation wavelengths in laser configuration were 405 nm, 488 nm, 546 nm and 633 nm. CompBeads (BD Biosciences) were used for single-color compensation to create multi-color compensation matrices. For gating, FMO controls were used. The instrument calibrated was controlled daily using Cytometer Setup and Tracking beads (BD Biosciences). Data were analyzed using FlowJo software v10.6.1 including the t-SNE plug-in.

### Hematoxylin and Eosin Staining (H&E Staining)

Paraformaldehyde-fixed mouse lung sections were deparaffinized, rehydrated with a series of xylol-ethanol-isopropanol solutions, washed with deionized water, incubated with Mayer’s hematoxylin solution (AP254766.1610; AppliChem GmbH, Darmstadt, Germany) for 10 min at room temperature, washed under running water for 5 min, and then incubated with eosin Y (AP253999.1210; AppliChem GmbH) for 2 min at room temperature followed by a short washing step with deionized water. After treatment with a series of ethanol-xylol solutions, the slides were mounted with Pertex mounting medium (CellPath Ltd., Newtownh, UK) and analyzed using a NanoZoomer digital slide scanner (Hamamatsu Photonics, Hamamatsu City, Japan).

### Statistics

Data were presented as as means ± SEM. Statistically significant differences between groups were identified using the one-way analysis with Dunnett’s test. Statistical analysis was performed with GraphPad Prism v8. Differences were considered significant if *, p ≤ 0.05, **, p ≤ 0.01, ***, p ≤ 0.001, ****, p ≤ 0.0001.

### Study Approval

All animal studies were approved by the local Ethics Committee (Regierungspräsidium Darmstadt, Hessen, Germany) and performed in accordance with the German Animal Welfare Act (Tierschutzgesetz).

## Data Availability Statement

The raw data supporting the conclusions of this article will be made available by the authors. Further inquiries can be directed to the corresponding authors.

## Ethics Statement

The animal study was reviewed and approved by Regierungspräsidium Darmstadt, Hessen, Germany.

## Author Contributions

CO, AW, SP, and RS conceptualized and designed research. CO and AW developed methodology. CO, DB, ÖA-C, ES-F, and AW performed experiments and acquired data. AW analyzed and interpreted results. SP, FG, WS, and BB provided technical and material support. AW and RS supervised research. All authors contributed to the article and approved the submitted version.

## Funding

This work was supported by Deutsche Krebshilfe (70112451), Deutsche Forschungsgemeinschaft (FOR 2438, TP8, SFB 1039, TP B04 and B06, GRK 2336, TP1 and 6, SFB 1213 TPA01, A05, and A10), Wilhelm-Sander Foundation (2019.082.01) and the LOEWE Center Frankfurt Cancer Institute (FCI) funded by the Hessen State Ministry for Higher Education, Research and the Arts [III L 5 - 519/03/03.001 - (0015)]. Further support was received from the Max Planck Society, Cardio-Pulmonary Institute (CPI), the German Center for Lung Research (DZL) and European Research Council (ERC) Consolidator Grant (#866051 to SSP).

## Conflict of Interest

The authors declare that the research was conducted in the absence of any commercial or financial relationships that could be construed as a potential conflict of interest.

## Publisher’s Note

All claims expressed in this article are solely those of the authors and do not necessarily represent those of their affiliated organizations, or those of the publisher, the editors and the reviewers. Any product that may be evaluated in this article, or claim that may be made by its manufacturer, is not guaranteed or endorsed by the publisher.

## Supplementary Material

Supplementary Figure S1 Gating tree for multispectral flow cytometry analysis of murine lung. Hierarchical analysis approach for pulmonary cell subsets showing the gating strategy with a multispectral antibody panel. BEC, blood endothelial cells; cDCs, conventional dendritic cells; LEC, lymphatic endothelial cells; MФ, macrophage; NK, natural killer cells; NKT, natural killer T cells; pDCs, plasmacytoid dendritic cell; Treg, regulatory T cell; VSMC, vascular smooth muscle cell.

Supplementary Figure S2 Quantification of cell subsets derived from CD45^+^ cells in different lung tumor models. Relative abundance of immune cell populations within the CD45^+^ cell compartment is shown. Control means healthy lung. i.t., intratracheal injection; i.v., intravenous injection; t.r., tumor relapse model; Macs, macrophages; Mono, monocytes; DCs, dendritic cells; pDCs, plasmacytoid dendritic cells; NK cells, natural killer cells; NKT cells, natural killer T cells; Treg, regulatory T cells. Animal numbers for control n=5, for i.t. model n=6, for *KRas* model n=9, for tumor relapse model n=9, for i.v. model n=3. *P < 0.05, **P < 0.01, ***P < 0.001, ****P < 0.0001 compared with control.
