# Supplementary figures and images for: Picturing of the Lung Tumor Cellular Composition by Multispectral Flow Cytometry
Source: Front Immunol. 2022 Jan 25;13:827719. doi: 10.3389/fimmu.2022.827719 (PMC8821098; doi:10.3389/fimmu.2022.827719)

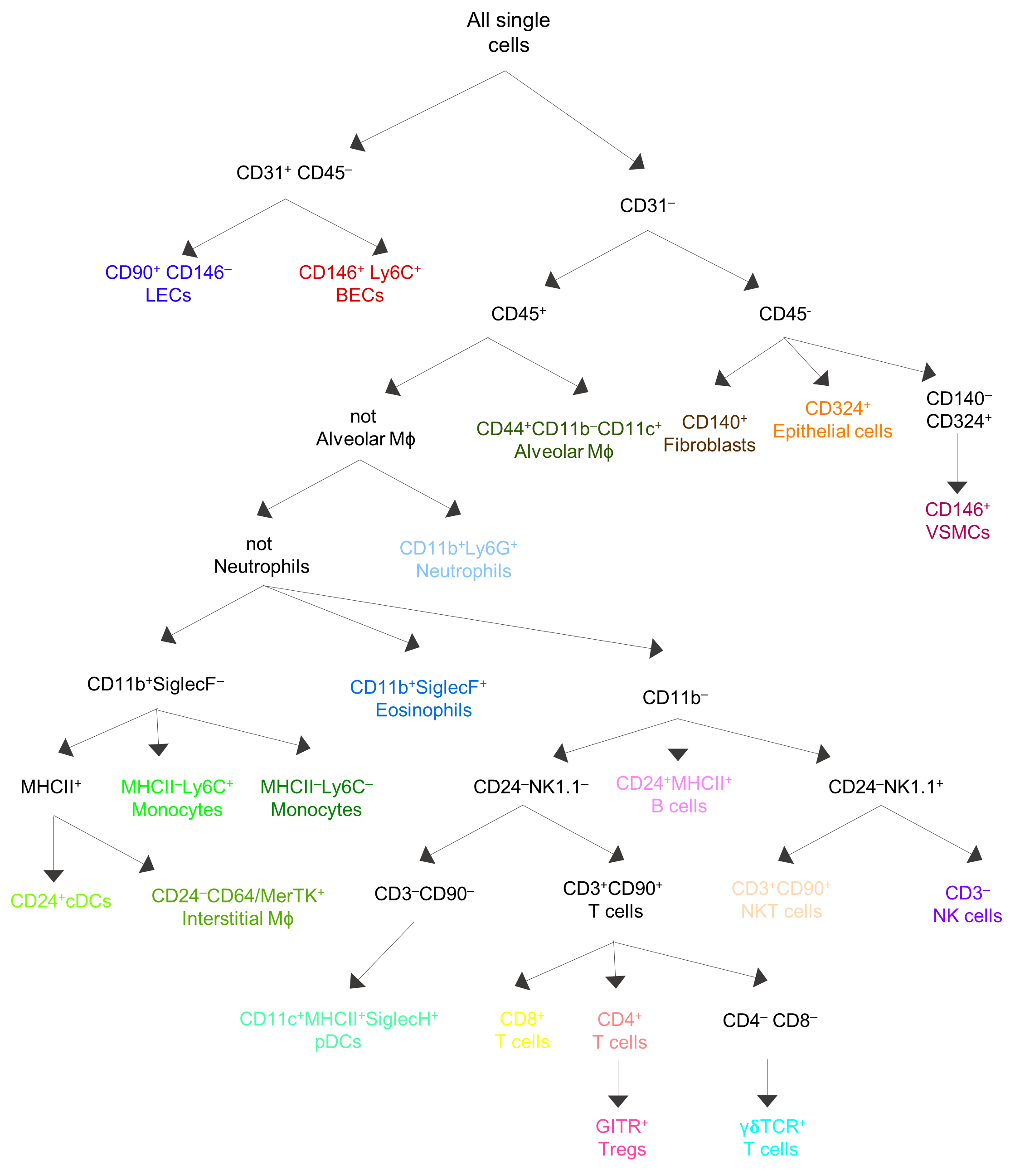

Supplement: Supplementary Figure S1 — Gating tree for multispectral flow cytometry analysis of murine lung. Hierarchical analysis approach for pulmonary cell subsets showing the gating strategy with a multispectral antibody panel. BEC, blood endothelial cells; cDCs, conventional dendritic cells; LEC, lymphatic endothelial cells; MФ, macrophage; NK, natural killer cells; NKT, natural killer T cells; pDCs, plasmacytoid dendritic cell; Treg, regulatory T cell; VSMC, vascular smooth muscle cell. [file Image_1.tif]

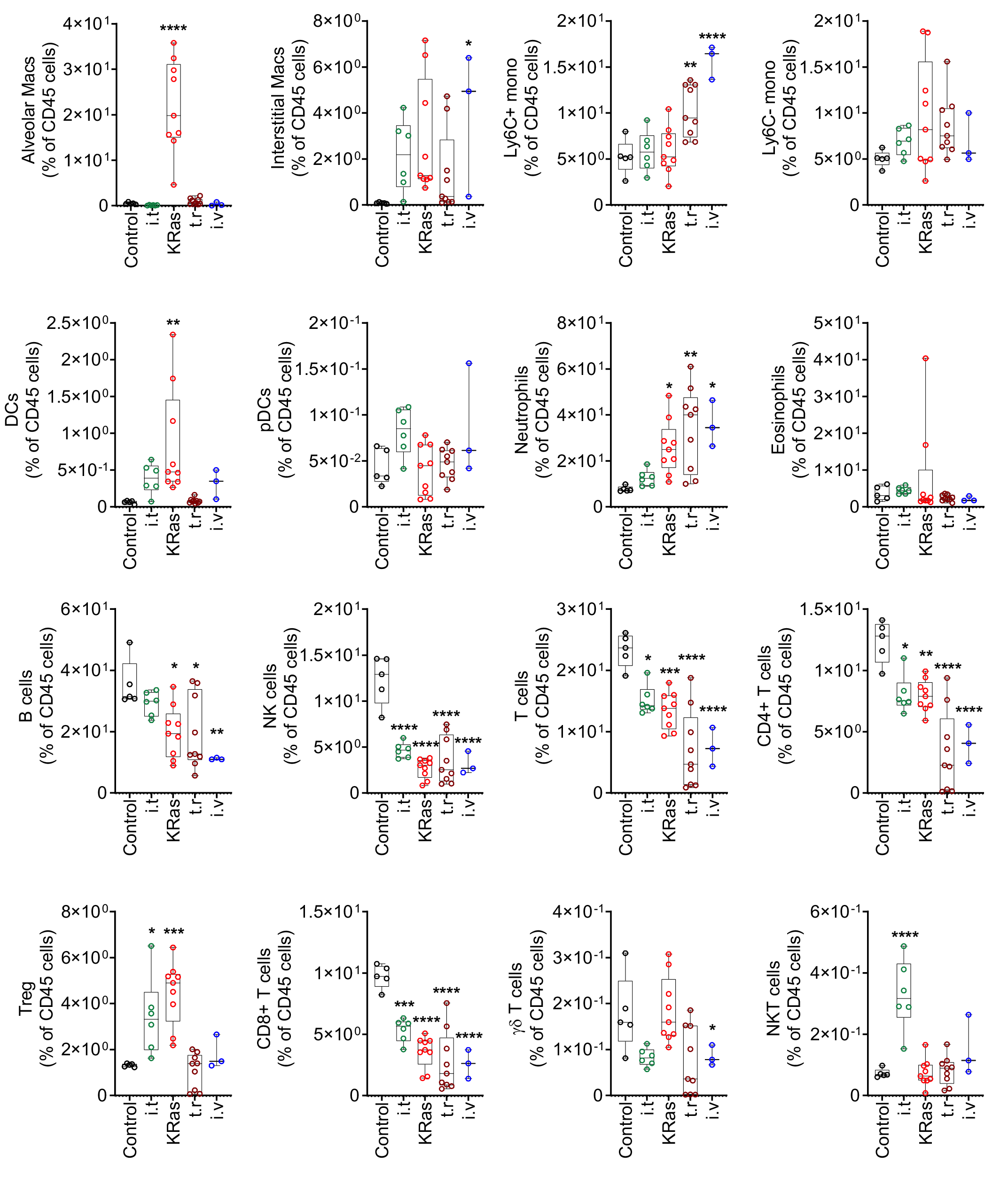

Supplement: Supplementary Figure S2 — Quantification of cell subsets derived from CD45+ cells in different lung tumor models. Relative abundance of immune cell populations within the CD45+ cell compartment is shown. Control means healthy lung. i.t., intratracheal injection; i.v., intravenous injection; t.r., tumor relapse model; Macs, macrophages; Mono, monocytes; DCs, dendritic cells; pDCs, plasmacytoid dendritic cells; NK cells, natural killer cells; NKT cells, natural killer T cells; Treg, regulatory T cells. Animal numbers for control n=5, for i.t. model n=6, for KRas model n=9, for tumor relapse model n=9, for i.v. model n=3. *P < 0.05, **P < 0.01, ***P < 0.001, ****P < 0.0001 compared with control. [file Image_2.tif]
